# How does mental health stigma get under the skin? Cross-sectional analysis using the Health Survey for England

**DOI:** 10.1016/j.ssmph.2019.100433

**Published:** 2019-06-13

**Authors:** C.L. Niedzwiedz

**Affiliations:** Institute of Health and Wellbeing, University of Glasgow, 1 Lilybank Gardens, Glasgow, G12 8RZ, United Kingdom

**Keywords:** Stigma, Biomarker, Mental health, Wellbeing, Quality of life, Allostatic load

## Abstract

Despite increased awareness of mental health problems, stigma persists. Little research has examined potential health and wellbeing outcomes associated with stigma. The aim of this study was to investigate relationships between mental health stigma, metabolic and cardiovascular biomarkers, as well as wellbeing and quality of life among people with no mental disorder, common mental disorders and severe mental illness. Data were taken from adults aged 16 + years participating in the Health Survey for England in 2014 (N = 5491). Mental health stigma was measured using the 12-item Community Attitudes towards the Mentally Ill (CAMI) scale, intended to measure attitudes around prejudice and exclusion, and tolerance and support for community care. Individuals were divided into six groups based on their mental health (no mental disorder, common mental disorder, severe mental illness) and whether they exhibited more (≤25^th^ percentile) or less (>25^th^ percentile) stigmatising attitudes. Metabolic and cardiovascular biomarker outcomes included systolic and diastolic blood pressure; total cholesterol; high-density lipoprotein (HDL) cholesterol; glycated haemoglobin, body mass index (BMI), waist-hip ratio and resting pulse rate. Biomarkers were analysed individually and as an allostatic load score. Wellbeing was measured using Warwick-Edinburgh Mental Wellbeing Scale (WEMWBS) and quality of life via Euro-QoL-5D (EQ-5D). Linear regression models were calculated adjusted for confounders. Compared to individuals with less stigmatising attitudes, results suggested that those with more negative attitudes exhibited poorer wellbeing and quality of life across all mental disorder/stigma groups, including those with no mental disorder (WEMWBS (range 14–70): b = -1.384, 95% CI: -2.107 to -0.661). People with severe mental illness generally had unhealthier biomarker profiles and allostatic load scores, but results were inconsistent for any additional influence of mental health stigma. Reducing stigma may be beneficial for population wellbeing, but further research is needed to clarify whether stigma contributes to adverse biomarkers amongst people with mental illness.

## Highlights

• Few studies have researched the consequences of mental health stigma.
• More stigmatising attitudes relate to poorer wellbeing amongst all groups.
• People with severe mental illness had more adverse biomarker profiles.
• Results were inconclusive for any additional influence of mental health stigma.

## 1 Background

Mental health disorders (such as major depressive disorder and anxiety disorders) are now a leading cause of disability worldwide (Vos et al., 2017). Substantial inequalities exist in life expectancy for people with severe mental illnesses (such as bipolar disorder and schizophrenia), quantified at 8.0 to 14.6 life years lost for men and 9.8 to 17.5 life years lost for women (Chang et al., 2011). Despite this, research that attempts to explain the premature and excess mortality and morbidity observed for those with mental disorders has received comparatively little attention, in contrast to other major risk factors such as diabetes and obesity. Stigma associated with mental illness is thought to be a key contributing factor (Saxena, 2018).

Stigma is a fundamental social determinant of health that leads to health inequalities (Hatzenbuehler, Phelan, & Link, 2013), yet there is a lack of research relating to the role of stigma in patterning health. This may be partly due to the inconsistent definition of stigma, the difficulty in measuring the concept and the many circumstances in which stigma has been used (Link & Phelan, 2001). Stigma can be understood as applying to a wide range of circumstances not limited to mental illness, such as welfare receipt (Besley & Coate, 1992), HIV and AIDS (Parker & Aggleton, 2003), and lung cancer (Chapple, Ziebland, & McPherson, 2004). Link and Phelan conceptualise stigma as the co-occurrence of several dimensions: labelling (when human differences are identified and named), stereotyping (dominant cultural beliefs connect labelled persons to undesirable characteristics), separation (labelled persons are grouped to achieve division of “us” versus “them”), status loss, and discrimination (Link & Phelan, 2001). They further stress that for stigmatisation to happen, power must be exercised and that stigma can lead to the unequal distribution of a variety of life chances including employment, housing and health (Link & Phelan, 2001). Within mental health, several ways in which stigma can be expressed have been distinguished: public stigma; internalised or self-stigma; and structural stigma.

Public stigma occurs when members of the general public endorse prejudice and discrimination against people with mental illness (Rusch & Thornicroft, 2014), such as believing people with mental disorders are highly dangerous. Self-stigma occurs when people with mental illness endorse and internalise these negative stereotypes, which can result in a loss of self-worth, shame, and lead individuals to give up on life goals (Rusch & Thornicroft, 2014), also known as the “why try” effect (Corrigan, Larson, & Rüsch, 2009). However, self-stigma is not inevitable. In different situations people with mental illness may respond to stigma with low self-esteem and diminished self-efficacy, righteous anger, or indifference (Corrigan & Watson, 2006). Some people may find their identity empowers them and that they can use their anger to improve their own circumstances and help others. Indeed, research has demonstrated that public stigma and self-stigma are correlated, but not necessarily strongly associated (Bradstreet, Dodd, & Jones, 2018; Chronister, Chou, & Liao, 2013). Structural, or institutional stigma, occurs when policies, rules or regulations within society intentionally marginalise the opportunities of those with mental disorders or produce unintended consequences that hinder their prospects, resources and wellbeing (Corrigan, Markowitz, & Watson, 2004; Hatzenbuehler, 2016; Rusch & Thornicroft, 2014). An example being the chronic under-funding of mental health services (Corrigan & Watson, 2003).

Given the lack of a consistent definition of stigma and its various forms, there is no consensus on how best to measure mental health stigma and numerous methods have been used (Link, Yang, Phelan, & Collins, 2004). A body of research has focussed on measuring public stigma towards people with mental disorders and its evolution over time. Data spanning ten years from 1996 to 2006 from the United States has revealed no decrease in public stigma, measured by asking respondents, for example, how willing they would be to have a person with a mental illness work closely with them, or marry into the family (Pescosolido et al., 2010). There was also a small increase in beliefs that people with schizophrenia would likely be violent towards others. This was supported by a study examining public attitudes across 8 years in Australia, where an increase in the perception that people with mental disorders are dangerous and unpredictable was observed (Reavley & Jorm, 2012). A systematic review on this topic supports these findings; despite improved population mental health literacy, the social rejection of people with mental disorders has remained pervasive over the last 20 years and negative stereotypes relating to the dangerousness of people with severe mental illness persists (Schomerus et al., 2012). In a study examining public attitudinal trends associated with the implementation of the Time to Change campaign to reduce mental health stigma and discrimination in England, Evans-Lacko, Henderson, and Thornicroft (2013) found little evidence for significant long-term improvements in knowledge and attitudes towards people with mental illness, or changes in reported behaviour from 2009 to 2012. However, there was some evidence to support improved intended behaviour, such as the intention to live, work and have a relationship with someone who has a mental illness. More recent evidence suggests progress has been made in reducing levels of public mental health stigma between 2009 and 2017 in England (Robinson & Henderson, 2018). Measures of public knowledge, attitudes, desire for social distance and reporting having contact with people with mental health problems have all shown improvements over time.

Few studies have adopted a multilevel approach to mental health stigma. One study that used data from 14 European countries found that people with mental illness who resided in countries with less stigmatising attitudes had lower rates of self-stigma and perceived discrimination (Evans-Lacko, Brohan, Mojtabai, & Thornicroft, 2012). Individuals who lived in countries where the public felt more comfortable interacting with people who had a mental illness also had lower levels of self-stigma and felt more empowered. In this study self-stigma was measured using the Internalised Stigma of Mental Illness Scale (ISMI), which contains alienation, stereotype endorsement, perceived discrimination and social withdrawal subscales.

Stigma has been associated with a range of outcomes amongst people with mental illness. A systematic review demonstrated a strong relationship between internalised stigma and poorer psychosocial outcomes (including hope, self-esteem and empowerment), as well as psychiatric symptom severity and poorer adherence to treatment (Livingston & Boyd, 2010). Increased depressive symptoms and poorer quality of life are also related to internalised stigma amongst those with mental disorders (Mashiach-Eizenberg, Hasson-Ohayon, Yanos, Lysaker, & Roe, 2013; Picco et al., 2016; Yanos, Roe, Markus, & Lysaker, 2008). Stigma may also contribute towards suicidality and suicide rates (Oexle, Waldmann, Staiger, Xu, & Rüsch, 2018; Rüsch, Zlati, Black, & Thornicroft, 2014; Schomerus et al., 2015), impede recovery from mental illness (Wood, Byrne, Burke, Enache, & Morrison, 2017), and hamper efforts to prevent mental disorders (Rusch & Thornicroft, 2014). Holding stigmatising beliefs about people with mental disorders is also related to less active help-seeking behaviour for mental ill health (Schnyder, Panczak, Groth, & Schultze-Lutter, 2017). However, a key gap in the literature relates to the lack of research focusing on possible objective health outcomes associated with self-stigma. A difficulty of measuring the potential health effects of self-stigma is the lack of available indicators included in large scale health surveys. An exception is the Health Survey for England (HSE), which included the Community Attitudes Towards the Mentally Ill scale (CAMI) in 2014, developed to measure public attitudes towards people with mental illness (Taylor & Dear, 1981). The scale has been used extensively to evaluate the Time to Change anti-stigma and discrimination campaign (Evans-Lacko et al., 2013). One way to measure aspects of self-stigma and its potential effects on health is to assess the extent of stereotype endorsement using the CAMI scale amongst people with mental disorders and relate this to the range of health outcomes included in the HSE.

To date, there have been no studies that have analysed the impact of mental health stigma on biological indicators of health, or biomarkers. A biomarker can be defined as “*a characteristic that is objectively measured and evaluated as an indicator of normal biological processes, pathogenic processes, or pharmacologic responses to a therapeutic intervention*” (Biomarkers Definitions Working Group, 2001, p. 91). This covers a variety of measures from pulse and blood pressure through to more complex laboratory tests of blood to assess levels of cholesterol or inflammatory markers (Strimbu & Tavel, 2010). Examining biomarker data has several advantages. Biomarkers are not affected by reporting biases as is the case for self-reported outcomes. They can help to identify individuals at an increased risk of health problems before people are aware of problems themselves, and elucidate potential causal mechanisms at play between social exposures and disease (Shih, Fernandes, & Bird, 2010). Given that self-stigma is associated with diminished self-esteem, poorer mental health and quality of life, it could be hypothesised to impact on biological indicators of health, particularly those related to cardiovascular and metabolic health. This has been demonstrated for other stressful experiences, such as informal caregiving (Lacey, McMunn, & Webb, 2018), financial insecurity (Niedzwiedz, Katikireddi, Reeves, McKee, & Stuckler, 2017), threat of redundancy (Mattiasson, Lindgärde, Nilsson, & Theorell, 1990), and household debt (Sweet, Nandi, Adam, & McDade, 2013). Potential stress associated with self-stigma, as well as perceived and anticipated discrimination (Schafer & Ferraro, 2011), may operate directly on metabolic and cardiovascular health via chronic physiological stress responses or through poor health behaviours, such as a diet characterised by high sugar and fat (Aschbacher et al., 2014), and a lack of physical activity.

The process by which stigma may affect biological systems could be considered an example of embodiment. Embodiment is “*a concept referring to how we literally incorporate, biologically, the material and social world in which we live, from conception to death*” (Krieger, 2001, p. 672). It is a useful construct to theorise how social exposures ‘get under the skin’, become biologically embedded and ultimately influence health and health inequalities (Krieger & Davey Smith, 2004). Social epidemiologists have drawn on embodiment to examine the potential biological pathways through which socioeconomic inequalities in health may arise, such as via the inflammatory system (McCrory et al., 2019) and allostatic load (Delpierre et al., 2016; McCrory et al., 2019). Allostatic load has been proposed as a measure of the overall cost of adapting to the environment and is usually operationalised as a composite measure including various physiological systems which represent physiological wear and tear (Delpierre et al., 2016). Markers included in allostatic load scores often comprise blood pressure, pulse rate, body mass index (BMI), and blood glucose, which are associated with metabolic and cardiovascular diseases, as well as all-cause mortality (Adams et al., 2009; Bhaskaran, dos-Santos-Silva, Leon, Douglas, & Smeeth, 2018; Palatini & Julius, 2004). The composite allostatic load scores have been found to predict mortality more accurately than the individual indicators themselves (Delpierre et al., 2016; Seeman, McEwen, Rowe, & Singer, 2001).

In this study, mental health stigma is conceptualised as a cumulative social exposure which can become embodied to impact on cardiovascular and metabolic function amongst people with mental illness. The study aims to advance the evidence base on the relationship between mental health stigma and health (measured by a range of metabolic and cardiovascular biomarkers and an indicator of allostatic load) in a general population sample. Previous research on the outcomes of mental health stigma has often used only self-reported psychological outcomes. To compare with previous research, measures of wellbeing and quality of life are also included. The research questions and specific hypotheses are detailed below:

1. What is the extent of mental health stigma amongst those with and without mental disorders and are there differences between individuals with severe and common mental disorders?
  • Individuals with mental disorders are hypothesised to display less stigmatising attitudes compared to those with no experience of mental illness.
2. Is mental health stigma associated with metabolic and cardiovascular biomarkers, wellbeing and quality of life and does any relationship differ between individuals with and without mental disorders?
  • Mental health stigma is not expected to relate to health and wellbeing in individuals with no diagnosed mental disorder. Individuals with mental disorders who hold stigmatising attitudes are hypothesised to have more adverse metabolic and cardiovascular biomarker profiles and poorer wellbeing and quality of life compared to those with no mental disorder and the associations may be stronger amongst those with more severe mental illness.

## 2 Methods

### 2.1 Data

Data were taken from the 2014 round of the Health Survey for England (HSE) (NatCen Social Research & University College London Department of Epidemiology and Public Health, 2018, p. 7919) and are available via the UK Data Service (https://www.ukdataservice.ac.uk/). The HSE is a repeated cross-sectional survey, which has been conducted annually since 1991. The sampling is based on a multi-stage stratified random sample of individuals living in private households in England. In the 2014 survey, the sample included 8077 adults (aged 16 years and over) and 2003 children (aged 0–15 years), with a household response rate of 62% (NatCen Social Research, 2014d). Each survey contains a range of health and sociodemographic related questions collected via face-to-face Computer Assisted Personal Interview (CAPI) and self-completion methods, as well as measurements taken by a nurse (including height, weight, waist circumference, blood pressure and urine, blood and saliva samples) at a follow-up visit for consenting participants. In addition, each year contains different modules focusing on a specific topic. The 2014 survey included a self-completion module dedicated to mental health, which was asked of adults only during the nurse visit. Participants were included in this study if they completed the follow-up nurse visit and were aged 16 years and over (N = 5491).

### 2.2 Independent variables

#### 2.2.1 Definition of mental disorder

During the nurse visit participants were provided with a list of 17 mental health disorders and asked to select which ones they had ever been diagnosed with by a health professional, at any point in their life (Bridges, 2015). Due to the low prevalence of specific disorders, they were grouped into common mental disorders (phobia, panic attacks, post-traumatic stress, generalised anxiety disorder, depression, post-natal depression or obsessive compulsive disorder), severe mental illnesses (bipolar disorder, eating disorder, nervous breakdown, personality disorder, psychosis or schizophrenia) or other complex mental illness (attention deficit hyperactivity disorder, attention deficit disorder, dementia, seasonal affective disorder, alcohol or drug dependence, or any other mental, emotional or neurological problem or condition). Respondents may have reported having more than one disorder and there is significant overlap across categories (Bridges, 2015). Participants were grouped into those who did not report a diagnosed mental disorder, those who reported a common mental disorder or other complex mental illness (due to the small number of participants in the latter group), and those who reported a severe mental illness. Individuals who reported having both a severe mental illness and a common mental disorder (or other complex mental illness) were classified as having a severe mental illness.

#### 2.2.2 Measurement of mental health stigma

Mental health stigma was measured using the Community Attitudes toward the Mentally Ill (CAMI) scale. The original CAMI questionnaire contained 40 statements relating to mental illness (e.g. “*it is frightening to think of people with mental problems living in residential neighbourhoods*” and “*mental illness is an illness like any other*”) (Taylor & Dear, 1981)NatCen Social Research, 2014b). The 2014 HSE contained a shortened 12-item scale containing items designed to measure mental health stigma and tolerance, which participants aged 16 + years were asked to self-complete (Ilic, Henderson, Henderson, Evans-Lacko, & Thornicroft, 2015). The 12-tem CAMI scale has been used in previous research evaluating the impact of the Time to Change social marketing campaign (Sampogna et al., 2017).

The HSE team conducted factor analysis on the scale, which revealed a two-factor structure relating to prejudice and exclusion and tolerance and support for community care. Participants were asked to rate how much they agreed or disagreed with each statement on a 5-point Likert Scale, which was scored as follows for positive statements: agree strongly (100), agree slightly (75), neither agree nor disagree (50), disagree slightly (25), disagree strongly (0). Participants were also given the option to answer “don’t know”, but these were excluded from the analysis. Negatively worded statements were reverse scored so that for each item, the mean scores ranged from 0 to 100, where a higher score corresponds to a more positive attitude (i.e. less prejudiced and more tolerant) (Ilic et al., 2015). A composite score (ranging from 0 to 100) was then calculated for each factor which was derived from the mean of the six items relating to each factor. Participants were included in the composite score if they answered (i.e. not responded “don’t know”) at least two of the six statements relating to each factor (Ilic et al., 2015). Two binary variables were then derived distinguishing those who scored ≤ the 25^th^ percentile on both CAMI scales as indicators of more stigmatising attitudes and those who scored > the 25^th^ percentile as less stigmatising attitudes. Amongst people with mental illness, the former variables therefore represent a key aspect of self-stigma, the endorsement of negative stereotypes.

### 2.3 Outcomes

Eight biomarkers were included as outcomes: glycated haemoglobin (HbA1c), total cholesterol, high-density lipoprotein (HDL) cholesterol, systolic and diastolic blood pressure, resting pulse rate, body mass index (BMI) and waist-hip ratio. Resting pulse rate, systolic and diastolic blood pressure are measures of cardiovascular function, whereas HbA1c, cholesterol, BMI and waist-hip ratio are indicators of metabolic function. Non-fasting blood samples were taken from participants at the time of the nurse visit and were sent to the labs at the Royal Victoria Hospital in Newcastle for analysis. Glycated haemoglobin (mmol/mol), total cholesterol (mmol/L) and HDL-cholesterol (mmol/L) values were derived by procedures outlined elsewhere (NatCen Social Research, 2014a). Systolic and diastolic blood pressure measurements (mmHg) were also taken from participants during the nurse visit using an Omron HEM 907 blood pressure monitor after the participants had been sitting quietly for 5 minutes (NatCen Social Research, 2014c). Three measurements were taken and the mean value of the second and third measurements was used. As recommended (Cui, Hopper, & Harrap, 2003; Lacey et al., 2018), 10 mmHg and 5 mmHg were added to the systolic blood pressures and diastolic blood pressures of individuals who reported they had taken antihypertensive medications in the past seven days, respectively. Resting pulse rate (bpm) was also recorded using the Omron HEM 907 three times, the first value was used due to the increase in pulse rate across the three measurements. In line with previous research, 1.18 mmol/L was added to total cholesterol if an individual reported taking statins, 4% was subtracted if they reported taking diuretics, 10% was added to HDL-cholesterol if they reported taking beta blockers and 1% was added to HbA1c if they reported taking insulin or any other anti-diabetic medications (Robertson et al., 2014, 2017). Measurements of height, weight, waist and hip circumference were also taken from participants, enabling the calculation of BMI (weight in kg/height in m^2^) and waist-hip ratio (waist circumference in cm/hip circumference in cm).

Additionally, for each of the eight biomarkers, participants were classified into sex-specific quartiles based on the distribution of scores. Individuals who fell into top quartile (for HbA1c, total cholesterol, BMI and waist-hip ratio, resting pulse rate, systolic and diastolic blood pressure) or bottom quartile (for HDL-cholesterol) were classed as ‘high risk’ and given a score of 1 and the remaining sample was given a score of 0. From that, a measure of allostatic load was calculated from the sum of each binary biomarker variable (ranging from 0 to 8). This method used to derive the allostatic load score has been used in numerous previous studies (McCrory et al., 2019; Robertson et al., 2017; Seeman et al., 2001). Individuals were included in the score if they had at least four complete biomarkers and excluded if they had missing values for more than four biomarkers. Sensitivity analyses excluding those missing more than four biomarkers did not affect the substantive results.

Two measures of wellbeing and quality of life were included. Mental wellbeing was measured using the Warwick-Edinburgh Mental Well-Being Scale (WEMWBS) (Tennant et al., 2007), in which participants are asked to tick the box that best describes their experience (e.g. feeling useful, relaxed and thinking clearly) over the last two weeks on a scale from none of the time, rarely, some of the time, often, or all of the time. Quality of life was measured using the EuroQol-5D (EQ-5D) scale, which asseses five dimensions: mobility; ability to carry out usual activities; self-care; pain/discomfort; anxiety/depression (EuroQol Group, 1990). Participants are asked to rate whether they had ‘no problem’, ‘some problem’ or an ‘extreme problem’ with each dimension. The HSE team converted answers to a single utility value based on a British EQ-5D scoring algorithm and weighted according to the social preference of the UK population (Søltoft, Hammer, & Kragh, 2009). For both WEMWBS (range 14–70) and EQ-5D (range -0.33 to 1.00) higher scores reflected more positive outcomes.

### 2.4 Covariates

Age in years, gender (male versus female), ethnicity (white versus non-white), marital/partnership status (married/cohabiting/civil partnership, single, divorced/separated, or widowed), education level and social class were included as potential confounding variables. Highest education level was categorised as degree level or equivalent; A Level or equivalent; General Certificate of Secondary Education (GCSE) or equivalent; no qualifications. Social class (household) was categorised as managerial and professional occupations; intermediate occupations; or routine and manual occupations (including households who were not currently employed), according to the National Statistics Socio-economic Classification (NS-SEC) three-category social class classification scheme.

### 2.5 Statistical analysis

Firstly, descriptive statistics of the key variables were examined in this cross-sectional analysis. Relevant weights were applied to account for non-response and selection into the different elements of the survey (e.g. the nurse visit and blood sample groups). Glycated haemoglobin values were logged due to their skewed distribution and when results from these models are presented their exponentiated coefficients are shown to help with the interpretation. First, the association between mental disorders (defined as a categorical variable comparing those with no mental disorder, a common mental disorder, or a severe mental illness) and mental health stigma (including the two attitudinal measures in turn as binary outcome variables) was assessed using logistic regression, adjusted for the covariates: age, gender, education level, social class, ethnicity and marital status. Next, the association between mental disorder, stigma and each biomarker and wellbeing outcome was examined using linear regression, adjusted for the covariates. Six mental disorder/stigma groups were derived: individuals with no mental disorder/less stigmatising attitudes (>25^th^ percentile); no mental disorder/more stigmatising attitudes (≤25^th^ percentile); common mental disorder/less stigmatising attitudes; common mental disorder/more stigmatising attitudes; severe mental disorder/less stigmatising attitudes; severe mental disorder/more stigmatising attitudes. The standardised beta coefficients from these models were also calculated and graphed to help interpret the pattern of results, effect sizes and direction of associations.

All statistical models adjusted for household clustering. Missing data for the independent variables and covariates (N = 524) were excluded from the analysis. Each statistical model may contain a different number of individuals as participants who had complete data for at least one outcome variable were included. Statistical analyses were conducted using Stata/MP 15.1.

## 3 Results

### 3.1 Sample description

A total of 4967 individuals were included in the analysis sample (excluding participants with missing data), 51.5% were female (Table 1). The mean age of participants was 46.7 (SD = 18.7). 73.2% (N = 3635) of the sample reported having no diagnosed mental disorder, 22.3% (N = 1110) a common mental disorder and 4.5% (N = 222) a severe mental illness. 32.2% (N = 1600) of the sample exhibited more stigmatising attitudes towards mental health according to the tolerance and support for community care measure, compared to 25.5% (N = 1267) using the prejudice and exclusion measure. Descriptive statistics for the outcome variables are found in Table A1.

**Table 1:** Descriptive statistics for the sample (weighted).

Variable	Mean	SD
**Age (years)**	46.7	18.7
**Gender**	**N**	**%**
Male	2409	48.5
Female	2558	51.5
**Mental disorder/stigma group (tolerance & support for community care)**	**N**	**%**
No MD/less stigmatising attitudes	2368	47.7
No MD/more stigmatising attitudes	1267	25.5
CMD/less stigmatising attitudes	837	16.9
CMD/more stigmatising attitudes	273	5.5
SMI/less stigmatising attitudes	162	3.3
SMI/more stigmatising attitudes	60	1.2
**Mental disorder/stigma group (prejudice & exclusion)**	**N**	**%**
No MD/less stigmatising attitudes	2611	52.6
No MD/more stigmatising attitudes	1025	20.6
CMD/less stigmatising attitudes	913	18.4
CMD/more stigmatising attitudes	197	4.0
SMI/less stigmatising attitudes	177	3.6
SMI/more stigmatising attitudes	45	0.9
**Education Level**	**N**	**%**
Degree	1395	28.1
A Level	1442	29.0
GCSE	1217	24.5
None	913	18.4
**Ethnicity**	**N**	**%**
White	4428	89.1
Non-white	539	10.9
**Marital status**	**N**	**%**
Single	1155	23.2
Married	3091	62.2
Divorced	425	8.6
Widowed	296	6.0
**Social class**	**N**	**%**
Managerial and professional occupations	2086	42.0
Intermediate occupations	1063	21.4
Routine and manual occupations	1818	36.6
**Total**	4967	100

CMD = common mental disorder; GCSE = General Certificate of Secondary Education; MD = mental disorder; N = number of individuals; SD = standard deviation; SMI = severe mental illness.

### 3.2 Mental health stigma

Individuals with experience of a common mental disorder or severe mental illness were less likely to exhibit stigmatising attitudes compared to those with no mental disorder (Table 2). Using the measure of tolerance and support for community care, individuals with a common mental disorder (OR = 0.648, 95% CI: 0.546 to 0.770) were slightly less likely to have stigmatising attitudes than those with a severe mental illness (OR = 0.669, 95% 0.465 to 0.963), but using the measure of prejudice and exclusion results were equivalent. Women were also less likely to hold stigmatising attitudes compared to men, as well as those with a more advantaged socioeconomic position, according to both education level and social class. Non-white ethnic groups exhibited more stigmatising attitudes particularly in relation to the measure of prejudice and exclusion.

**Table 2:** Results from logistic regression models assessing the likelihood of more stigmatising attitudes (≤25^th^ percentile) according to mental disorder group.

	Tolerance & support for community care	Prejudice & exclusion
	OR [95% CI]	OR [95% CI]
**Gender (ref** = **male)**		
Female	0.765^∗∗∗^ [0.667,0.877]	0.588^∗∗∗^ [0.503,0.686]
**Age**	0.983^∗∗∗^ [0.978,0.988]	1.004 [0.999,1.010]
**Mental disorder (ref** = **none)**		
Common mental disorder	0.648^∗∗∗^ [0.546,0.770]	0.627^∗∗∗^ [0.519,0.756]
Severe mental illness	0.669^∗^ [0.465,0.963]	0.627^∗^ [0.437,0.898]
**Education level (ref** = **degree)**		
A Level	1.342^∗∗^ [1.083,1.663]	1.117 [0.874,1.427]
GCSE	1.494^∗∗∗^ [1.192,1.872]	1.418^∗∗^ [1.101,1.826]
None	1.944^∗∗∗^ [1.502,2.515]	2.311^∗∗∗^ [1.755,3.045]
**Ethnicity (ref** = **white)**		
Non-white	1.523^∗∗^ [1.153,2.011]	3.047^∗∗∗^ [2.279,4.075]
**Marital status (ref** = **married)**		
Single	1.072 [0.866,1.328]	0.927 [0.728,1.181]
Divorced	1.076 [0.846,1.368]	1.194 [0.935,1.524]
Widowed	1.269 [0.960,1.677]	1.470^∗∗^ [1.109,1.950]
**Social class (ref** = **Managerial & professional)**		
Intermediate	1.435^∗∗∗^ [1.162,1.771]	1.446^∗∗^ [1.149,1.821]
Routine and manual	1.589^∗∗∗^ [1.303,1.938]	2.073^∗∗∗^ [1.677,2.564]

^∗^*p* < 0.05.

^∗∗^*p* < 0.01.

^∗∗∗^*p* < 0.001.

CI = confidence interval; GSCE = General Certificate of Secondary Education; OR = odds ratio; Ref = reference category.

### 3.3 Metabolic and cardiovascular biomarkers

**Fig. 1:**
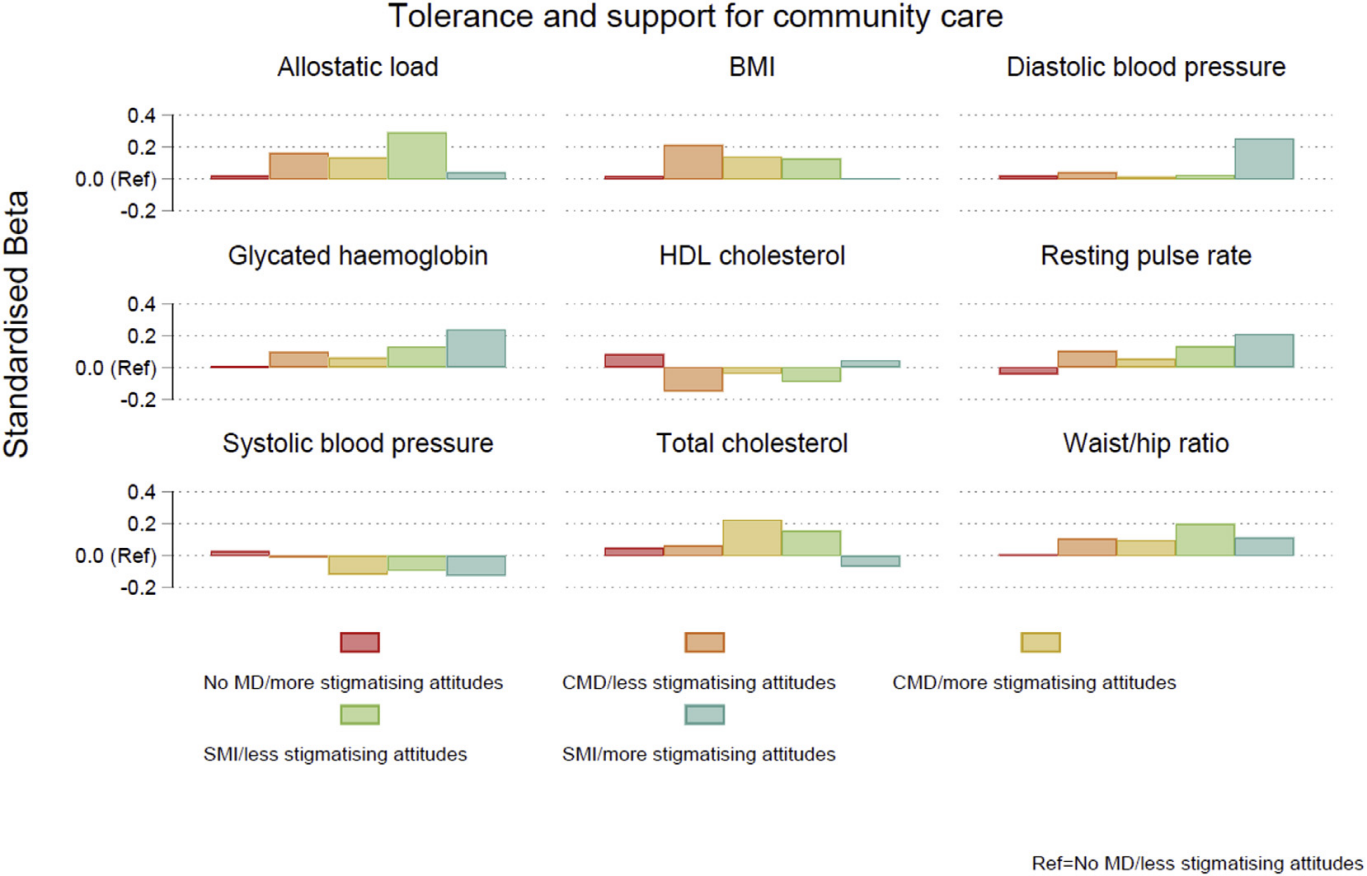
Standardised beta coefficients derived from linear regression models for each biomarker according to mental disorder/stigma group for the tolerance and support for community care measure. Note: BMI=Body Mass Index; CMD = common mental disorder; HDL = high density lipoprotein; MD=mental disorder; SMI = severe mental illness.

**Fig. 2:**
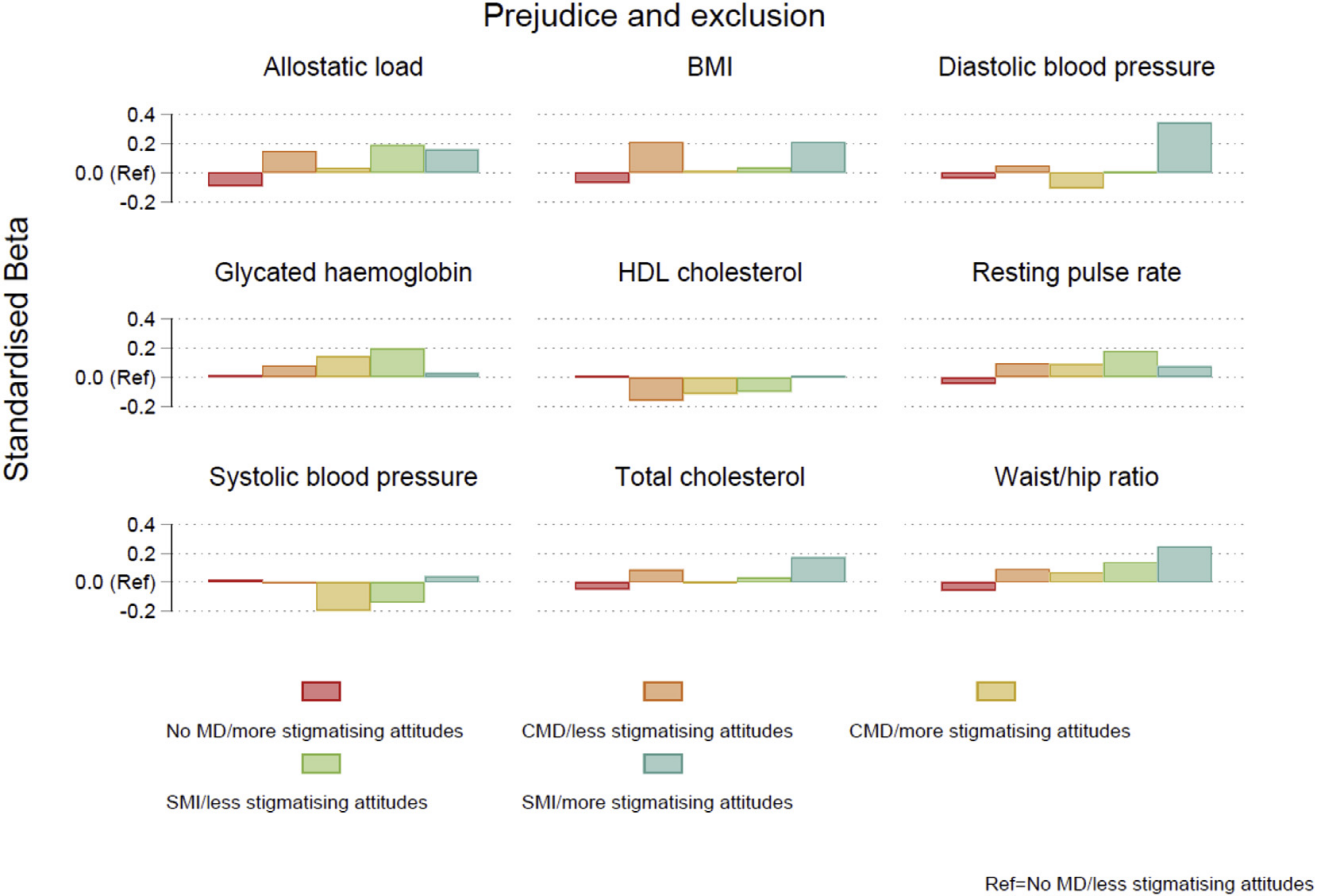
Standardised beta coefficients derived from linear regression models for each biomarker according to mental disorder/stigma group for the prejudice and exclusion measure. Note: BMI=Body Mass Index; CMD = common mental disorder; HDL = high density lipoprotein; MD=mental disorder; SMI = severe mental illness.

A mixed pattern of results was found for the metabolic and cardiovascular biomarkers (Fig. 1 and Fig. 2). Inconclusive results were found for the cardiovascular biomarkers, systolic and diastolic blood pressure (Table 3, full results available in Tables A2 and A3). Individuals with severe mental illness generally displayed higher resting pulse rates compared to those with a common mental disorder, and those with a common mental disorder exhibited higher values compared to those with no disorder. However, there were no notable differences dependent on the degree of mental health stigma possessed. Similar results were found for the metabolic biomarkers. For waist-hip ratio, glycated haemoglobin, and cholesterol, in general, more adverse biomarker levels were found with increased severity of mental illness, but little consistent differences were observed between stigma groups. Amongst those with severe mental illness, those with more stigmatising attitudes according to the measure of tolerance and support for community care exhibited higher levels of glycated haemoglobin (exponentiated coefficient = 1.042, 95% CI: 0.986 to 1.101), compared to those with less stigmatising attitudes (1.023, 95% CI: 0.985 to 1.062), but this was not found for the other measure of stigma and differences were not statistically significant. Likewise, for waist-hip ratio and BMI, higher values were observed for those with severe mental illness who displayed more stigmatising attitudes, compared to those with less stigmatising attitudes, but this was only observed for the measure of stigma related to prejudice and exclusion.

**Table 3:** Results from linear regression models assessing the association between mental disorder/stigma group and metabolic and cardiovascular biomarkers.

	Glycated haemoglobin^a^	Total cholesterol (mmol/L)	HDL cholesterol (mmol/L)	Systolic blood pressure (mmHg)	Diastolic blood pressure (mmHg)	Resting pulse rate (bpm)	Waist/hip ratio	BMI (kg/m^2^)	Allostatic load score
	Coeff. [95% CI]	Coeff. [95% CI]	Coeff. [95% CI]	Coeff. [95% CI]	Coeff. [95% CI]	Coeff. [95% CI]	Coeff. [95% CI]	Coeff. [95% CI]	Coeff. [95% CI]
**Tolerance & support for community care**^b^									
No MD/more stigmatising attitudes	1.000 [0.986,1.014]	0.057 [-0.050,0.163]	0.038 [-0.003,0.080]	0.567 [-0.730,1.863]	0.244 [-0.756,1.244]	-0.496 [-1.445,0.452]	0.001 [-0.005,0.006]	0.099 [-0.327,0.525]	0.037 [-0.102,0.176]
CMD/less stigmatising attitudes	1.017^∗^ [1.000,1.033]	0.073 [-0.035,0.181]	-0.068^∗∗∗^ [-0.109,-0.028]	-0.234 [-1.593,1.124]	0.478 [-0.497,1.453]	1.178^∗^ [0.199,2.157]	0.010^∗∗^ [0.004,0.016]	1.168^∗∗∗^ [0.685,1.650]	0.269^∗∗∗^ [0.125,0.413]
CMD/more stigmatising attitudes	1.011 [0.991,1.031]	0.247^∗∗^ [0.089,0.405]	-0.018 [-0.082,0.046]	-2.190^∗^ [-4.286,-0.094]	0.180 [-1.375,1.734]	0.605 [-0.749,1.959]	0.009 [-0.001,0.019]	0.760 [-0.047,1.567]	0.221 [-0.016,0.458]
SMI/less stigmatising attitudes	1.023 [0.985,1.062]	0.174 [-0.065,0.414]	-0.040 [-0.121,0.041]	-1.716 [-4.678,1.247]	0.291 [-2.004,2.586]	1.553 [-0.522,3.629]	0.018^∗^ [0.003,0.034]	0.705 [-0.334,1.743]	0.475^∗^ [0.098,0.852]
SMI/more stigmatising attitudes	1.042 [0.986,1.101]	-0.072 [-0.357,0.213]	0.019 [-0.150,0.189]	-2.295 [-6.792,2.202]	2.824 [-0.965,6.612]	2.393 [-1.215,6.001]	0.011 [-0.011,0.033]	0.024 [-1.802,1.850]	0.069 [-0.524,0.661]
**Prejudice & exclusion**^b^									
No MD/more stigmatising attitudes	1.003 [0.986,1.020]	-0.053 [-0.173,0.066]	0.002 [-0.043,0.046]	0.362 [-1.110,1.833]	-0.456 [-1.536,0.623]	-0.487 [-1.501,0.526]	-0.005 [-0.011,0.001]	-0.380 [-0.819,0.060]	-0.151^∗^ [-0.295,-0.006]
CMD/less stigmatising attitudes	1.014 [0.999,1.029]	0.097 [-0.004,0.199]	-0.072^∗∗∗^ [-0.112,-0.032]	-0.146 [-1.447,1.155]	0.534 [-0.399,1.467]	1.088^∗^ [0.164,2.011]	0.008^∗∗^ [0.002,0.015]	1.131^∗∗∗^ [0.657,1.606]	0.239^∗∗∗^ [0.101,0.378]
CMD/more stigmatising attitudes	1.026^∗^ [1.002,1.051]	0.003 [-0.204,0.211]	-0.052 [-0.118,0.014]	-3.608^∗∗^ [-5.874,-1.343]	-1.225 [-2.872,0.421]	1.048 [-0.663,2.759]	0.006 [-0.004,0.016]	0.076 [-0.737,0.889]	0.051 [-0.227,0.329]
SMI/less stigmatising attitudes	1.035 [0.998,1.074]	0.038 [-0.169,0.244]	-0.045 [-0.131,0.042]	-2.560 [-5.397,0.277]	0.056 [-2.215,2.327]	2.067 [-0.016,4.151]	0.013 [-0.002,0.027]	0.194 [-0.769,1.157]	0.308 [-0.064,0.680]
SMI/more stigmatising attitudes	1.005 [0.954,1.060]	0.188 [-0.276,0.651]	0.003 [-0.153,0.159]	0.715 [-3.738,5.168]	3.826^∗^ [0.810,6.842]	0.858 [-2.621,4.337]	0.022 [-0.002,0.046]	1.139 [-0.904,3.183]	0.259 [-0.274,0.792]
N	3555	3429	3559	4239	4239	4875	4762	4605	3680

BMI=Body Mass Index; CI = confidence interval; CMD = common mental disorder; Coeff. = unstandardised coefficient; HDL = high density lipoprotein; MD = mental disorder; N = number of individuals; SMI = severe mental illness.

^∗^*p* < 0.05.

^∗∗^*p* < 0.01.

^∗∗∗^*p* < 0.001.

All models adjusted for gender, age, education level, ethnicity, social class and marital status.

a Exponentiated coefficient.

b Reference category is no MD/less stigmatising attitudes.

### 3.4 Allostatic load

No clear pattern of results was found for allostatic load (Figs. 1 and 2). Individuals with common mental disorders generally exhibited higher allostatic load scores compared to those with no history of mental disorder, and those with severe mental illness had higher scores than those with a common mental disorder (Table 3, full results available in Tables A2 and A3). For example, amongst individuals displaying less stigmatising attitudes according to the measure of stigma relating to tolerance and support for community care, those with a common mental disorder had higher allostatic load scores compared to those with no disorder (b = 0.269, 95% CI: 0.125 to 0.413), and those with a severe mental illness had even higher scores (b = 0.475, 95% CI: 0.098 to 0.852). However, no consistent differences were apparent between those with higher and lower levels of mental health stigma.

### 3.5 Wellbeing and quality of life

**Fig. 3:**
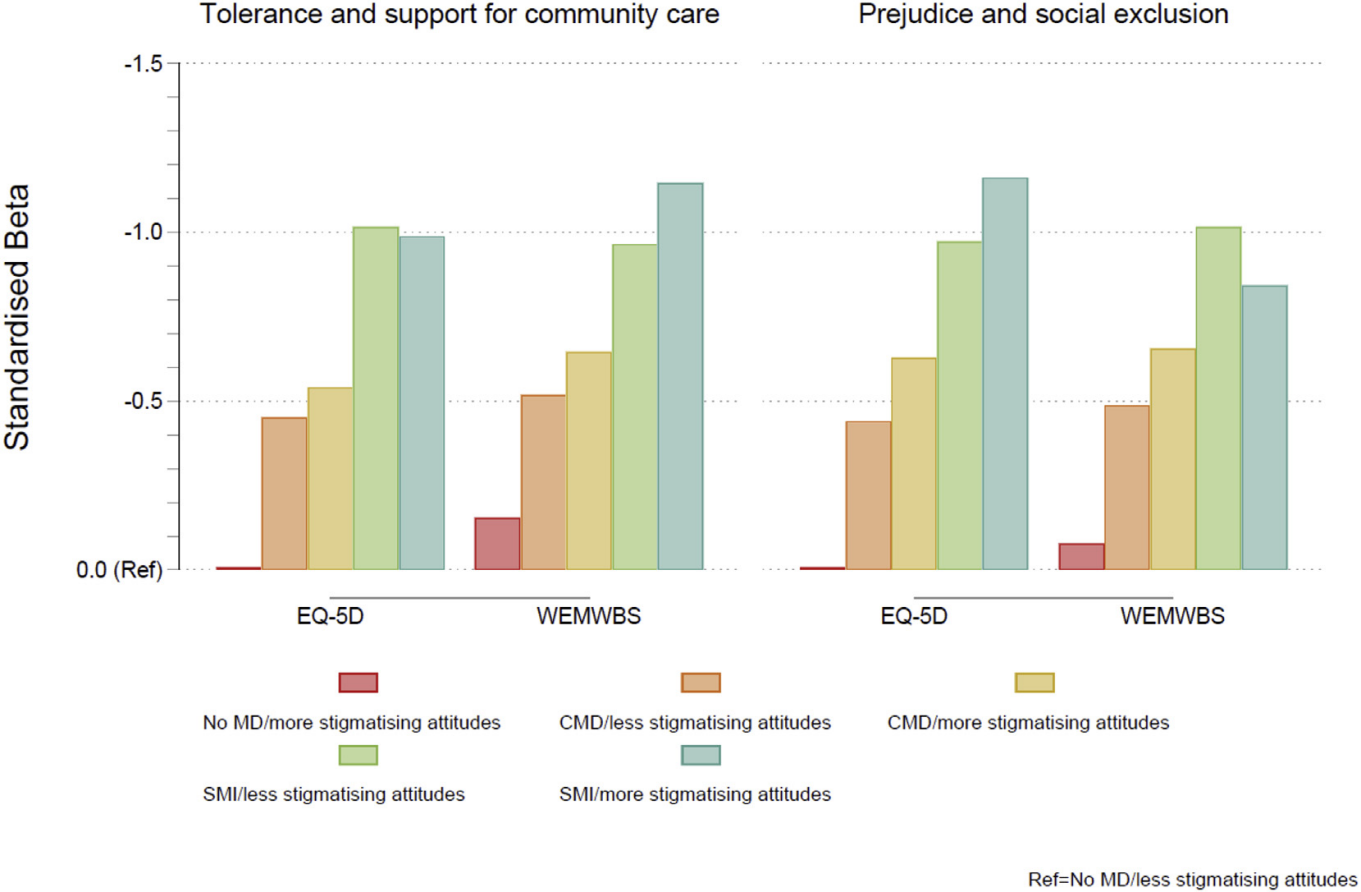
Standardised beta coefficients derived from linear regression models for wellbeing and quality of life according to mental disorder/stigma group. Note: CMD = common mental disorder; MD = mental disorder; SMI = severe mental illnes.

A clearer pattern of results was found for the measures of wellbeing and quality of life (Fig. 3). Compared to individuals with less stigmatising attitudes, those with more stigmatising attitudes generally exhibited poorer scores across all mental disorder/stigma groups (Table 4, full results available in Tables A2 and A3). Two exceptions were found amongst those with severe mental illness. Those who exhibited more stigmatising attitudes had slightly better quality of life when stigma was measured using the tolerance and support for community care indicator and slightly better wellbeing when using the prejudice and exclusion indicator, compared to those exhibiting less stigmatising attitudes. It was interesting to note that, when using the tolerance and support for community care indicator of stigma, worse wellbeing was apparent among those with more stigmatising attitudes compared to those with less stigmatising attitudes even amongst those with no experience of a mental disorder (b = -1.384, 95% CI: -2.107 to -0.661).

**Table 4:** Results from linear regression models assessing the association between mental disorder/stigma group and wellbeing and quality of life.

	WEMWBS	EQ5D	WEMWBS	EQ5D
	Coeff. [95% CI]	Coeff. [95% CI]	Coeff. [95% CI]	Coeff. [95% CI]
	Tolerance & support for community care a		Prejudice & exclusion a	
No MD/more stigmatising attitudes	-1.384^∗∗∗^ [-2.107,-0.661]	-0.002 [-0.016,0.013]	-0.688 [-1.558,0.183]	-0.002 [-0.017,0.014]
CMD/less stigmatising attitudes	-4.661^∗∗∗^ [-5.426,-3.895]	-0.106^∗∗∗^ [-0.126,-0.086]	-4.373^∗∗∗^ [-5.101,-3.645]	-0.103^∗∗∗^ [-0.122,-0.084]
CMD/more stigmatising attitudes	-5.798^∗∗∗^ [-6.988,-4.608]	-0.127^∗∗∗^ [-0.161,-0.092]	-5.897^∗∗∗^ [-7.302,-4.493]	-0.147^∗∗∗^ [-0.189,-0.105]
SMI/less stigmatising attitudes	-8.670^∗∗∗^ [-10.478,-6.862]	-0.238^∗∗∗^ [-0.294,-0.183]	-9.140^∗∗∗^ [-10.912,-7.367]	-0.228^∗∗∗^ [-0.279,-0.177]
SMI/more stigmatising attitudes	-10.312^∗∗∗^ [-13.554,-7.070]	-0.232^∗∗∗^ [-0.325,-0.138]	-7.579^∗∗∗^ [-11.262,-3.896]	-0.272^∗∗∗^ [-0.389,-0.155]
N	4792	4871	4792	4871

CI = confidence interval; CMD = common mental disorder; Coeff. = unstandardised coefficient; EQ5D = EuroQol-5D; MD = mental disorder; N = number of individuals; SMI = severe mental illness; WEMWBS=Warwick-Edinburgh Mental Well-Being Scale.

^∗^*p* < 0.05.

^∗∗^*p* < 0.01.

^∗∗∗^*p* < 0.001.

All models adjusted for gender, age, education level, ethnicity, social class and marital status.

a Reference category is no MD/less stigmatising attitudes.

## 4 Discussion

### 4.1 Summary

This study is the first to investigate the association between mental health stigma and a range of metabolic and cardiovascular biomarkers, alongside measures of wellbeing and quality of life in a general population sample. Less stigmatising attitudes were found amongst those with experience of mental ill health. A potential negative influence of mental health stigma was suggested for the measures of wellbeing and quality of life. Even for those with no mental disorder, individuals with more stigmatising attitudes had lower wellbeing compared to those with more positive attitudes and there was some indication that wellbeing and quality of life were worse amongst those with more stigmatising attitudes in each mental disorder group. The results for the metabolic and cardiovascular biomarkers were less convincing and often differed depending on the measure of stigma being used. There was evidence that those with more severe mental illness had more adverse levels of several biomarkers compared to those with a common mental disorder, and those with a common mental disorder generally had a better biomarker profile compared to those with no history of mental disorder. However, results were inconsistent for any additional influence of mental health stigma. Similarly, findings for allostatic load were mixed with regards to mental health stigma, but individuals with experience of a mental disorder had higher scores compared to those with no history.

Previous research has demonstrated that mental health stigma is related to wellbeing, life satisfaction and quality of life among people with mental illness (Markowitz, 1998; Park, Bennett, Couture, & Blanchard, 2013; Picco et al., 2016). Our findings add to and expand on this literature, suggesting that more stigmatising attitudes relate to poorer wellbeing and quality of life amongst those with mental disorders and associations may be stronger amongst those with severe mental illness. A novel finding of this paper which has not been examined before relates to the lower levels of wellbeing amongst those with no history of mental disorder who hold more stigmatising attitudes, compared to people who hold more positive attitudes. One contributing factor that merits further research may be mental health literacy, which has been shown to relate to higher wellbeing, and may influence help-seeking behaviour and positive coping skills (Bjørnsen, Espnes, Eilertsen, Ringdal, & Moksnes, 2017; Gulliver, Griffiths, & Christensen, 2010; Riebschleger, Costello, Cavanaugh, & Grové, 2019).

### 4.2 Strengths and limitations

A key strength of this study was the use of nationally representative data for England, obtained via the Health Survey for England. The analysis also used several different measures of metabolic and cardiovascular function and two measures of wellbeing and quality of life, as well as two indicators of mental health stigma, which are widely used and validated measures. The definition of mental health disorders also focused on lifetime diagnosed conditions, which is an improvement on some studies which often define mental ill-health based on a cut-off point using a scale measuring recent psychological distress, such as the General Health Questionnaire (Katikireddi, Niedzwiedz, & Popham, 2012). The analysis also included a range of potential confounding factors, although the possibility of unmeasured confounding cannot be eliminated.

This study has a few limitations that should be acknowledged. The definition of a mental disorder was based on self-reported diagnosis, which itself could be affected by stigma. Stigma could influence the disclosure of a diagnosed mental disorder within the survey and some individuals may have experienced mental ill health, but not sought a diagnosis. Although a validated measure of mental health stigma was used, answers to the questionnaire may be subject to social desirability bias (Henderson, Evans-Lacko, Flach, & Thornicroft, 2012). The CAMI also does not measure personal experience of self-stigma, such as the experience of shame and discrimination related to mental health disorders, which has been found to strongly associate with comorbid depression and anxiety (Alonso et al., 2008). It may be possible that different results may be obtained depending on the measure of stigma used. The measure of mental health stigma used in this study is intended to measure public stigma and self-stigma was implied via low CAMI scores (i.e. the endorsement of stigmatising stereotypes) amongst those who experienced a mental disorder. It does not measure the internalisation of stigmatising beliefs; it is possible to hold stigmatising attitudes towards other people with mental disorders but not apply or internalise them personally.

People at the most severe end of mental illness may be less likely to participate in health surveys and stigma may affect participation in surveys, the choice to complete the mental health questionnaires, and the answers provided (Woodall, Morgan, Sloan, & Howard, 2010). In addition, some of the included analyses comparing differences between severe and common mental disorders were underpowered due to the small number of people with a severe mental illness. Reverse causation also cannot be ruled out especially for the measures of wellbeing as those with poorer mental health may attribute this to stigma. The cross-sectional design of the study also precludes any inference of potential causal effects; longitudinal data are needed to investigate the research questions in more depth. At present, there are a lack of longitudinal data collected on mental health stigma and even fewer which also collect biomarker health data. The measure of allostatic load used in this study also only covered metabolic and cardiovascular function.

### 4.3 Conclusions

This study highlights the need for more research into the potential relationships between stigma, health and wellbeing. It is likely that multiple stigma processes operate in a complex manner. This includes stigma related to mental health, but also associated with other minority and disadvantaged statuses related to, for example, gender, ethnicity, sexual orientation, socioeconomic position, physical illness and disabilities. Therefore, future research would benefit from taking an intersectional approach to stigma to analyse how different stigmatised statuses interact to influence health and health inequalities. There is also a need to consider stigma at multiple levels (e.g. self-stigma, public stigma and structural stigma) and how these might interact to influence individual and population health. Longitudinal research that adopts a life course perspective and examines the evolution of mental health stigma through time within the same individuals to investigate whether there are particular critical periods in the life course (e.g. adolescence) that matter more for future health and social outcomes would also be valuable.

## Conflicts of interest

None.

## Ethics

No ethical approval was required as the study is an analysis of secondary data. Ethical approval for the Health Survey for England was obtained by the survey team.
