# Supplementary material for: How does mental health stigma get under the skin? Cross-sectional analysis using the Health Survey for England
Source: SSM Popul Health. 2019 Jun 13;8:100433. doi: 10.1016/j.ssmph.2019.100433 (PMC6609872; doi:10.1016/j.ssmph.2019.100433)
Supplement: Multimedia component 1 [file mmc1.docx]

Table A.1: Descriptive statistics for the outcome variables (weighted)

| **Outcome** | **N** | **Mean** | **SD** |
| --- | --- | --- | --- |
| Glycated haemoglobin | 3555 | 3.60 | 0.17 |
| Total cholesterol (mmol/L) | 3429 | 5.10 | 1.10 |
| HDL cholesterol (mmol/L) | 3559 | 1.54 | 0.44 |
| Systolic blood pressure (mmHg) | 4239 | 126.29 | 17.92 |
| Diastolic blood pressure (mmHg) | 4239 | 73.23 | 11.32 |
| Resting pulse rate (bpm) | 4875 | 69.66 | 11.45 |
| Waist-hip ratio | 4762 | 0.87 | 0.09 |
| Body mass index | 4605 | 27.13 | 5.43 |
| Allostatic load score | 3680 | 1.78 | 1.60 |
| WEMWBS | 4792 | 50.81 | 9.02 |
| EQ-5D | 4871 | 0.86 | 0.22 |

EQ-5D=EuroQol-5D; HDL=high density lipoprotein; N=number of individuals; SD=standard deviation; WEMWBS=Warwick-Edinburgh Mental Well-Being Scale

Table A.2: Results from linear regression models assessing the association between mental disorder/stigma group and health and wellbeing outcomes for the measure of tolerance and support for community care

|  | **Glycated haemoglobin**^a^ | **Total cholesterol (mmol/L)** | **HDL cholesterol (mmol/L)** | **Systolic blood pressure (mmHg)** | **Diastolic blood pressure (mmHg)** | **Resting pulse rate (bpm)** | **Waist-hip ratio** | **BMI (kg/m^2^)** | **Allostatic load score** | **WEMWBS** | **EQ-5D** |
| --- | --- | --- | --- | --- | --- | --- | --- | --- | --- | --- | --- |
|  | Coeff.  [95% CI] | Coeff.  [95% CI] | Coeff.  [95% CI] | Coeff.  [95% CI] | Coeff.  [95% CI] | Coeff.  [95% CI] | Coeff.  [95% CI] | Coeff.  [95% CI] | Coeff.  [95% CI] | Coeff.  [95% CI] | Coeff.  [95% CI] |
| **Gender (ref=male)** |  |  |  |  |  |  |  |  |  |  |  |
| Female | 0.996 [0.985,1.007] | 0.056 [-0.030,0.141] | 0.282^***^ [0.251,0.313] | -8.183^***^ [-9.209,-7.156] | -1.210^**^ [-1.961,-0.459] | 2.917^***^ [2.174,3.659] | -0.094^***^ [-0.098,-0.089] | -0.152 [-0.481,0.176] | -0.074 [-0.180,0.033] | 0.249 [-0.312,0.810] | -0.011 [-0.023,0.001] |
| **Age** | 1.004^***^ [1.003,1.004] | 0.014^***^ [0.011,0.017] | 0.003^***^ [0.002,0.004] | 0.428^***^ [0.391,0.464] | 0.103^***^ [0.075,0.131] | -0.031^*^ [-0.057,-0.005] | 0.002^***^ [0.002,0.002] | 0.055^***^ [0.042,0.068] | 0.031^***^ [0.027,0.035] | 0.017 [-0.004,0.038] | -0.002^***^ [-0.003,-0.002] |
| **Education level (ref=degree)** |  |  |  |  |  |  |  |  |  |  |  |
| A Level | 1.013 [0.999,1.027] | 0.002 [-0.109,0.114] | -0.084^***^ [-0.126,-0.042] | 1.679^*^ [0.272,3.085] | 0.227 [-0.851,1.306] | 0.276 [-0.768,1.319] | 0.007^*^ [0.001,0.014] | 0.315 [-0.138,0.769] | 0.231^**^ [0.092,0.370] | -0.447 [-1.195,0.301] | -0.008 [-0.023,0.006] |
| GCSE | 1.030^***^ [1.014,1.047] | 0.056 [-0.066,0.177] | -0.100^***^ [-0.148,-0.052] | 0.868 [-0.626,2.361] | -0.439 [-1.620,0.743] | 0.944 [-0.190,2.079] | 0.011^**^ [0.004,0.018] | 1.123^***^ [0.592,1.654] | 0.354^***^ [0.185,0.522] | -1.576^***^ [-2.394,-0.758] | -0.033^***^ [-0.049,-0.016] |
| None | 1.038^***^ [1.019,1.059] | -0.191^*^ [-0.337,-0.045] | -0.106^***^ [-0.165,-0.046] | 1.419 [-0.441,3.279] | -3.090^***^ [-4.445,-1.734] | 1.237 [-0.075,2.549] | 0.012^**^ [0.004,0.021] | 0.673^*^ [0.029,1.316] | 0.231^*^ [0.038,0.424] | -2.600^***^ [-3.643,-1.558] | -0.072^***^ [-0.095,-0.050] |
| **Ethnicity (ref=white)** |  |  |  |  |  |  |  |  |  |  |  |
| Non-white | 1.066^***^ [1.038,1.095] | -0.089 [-0.264,0.086] | -0.089^**^ [-0.150,-0.027] | -2.781^**^ [-4.596,-0.965] | 0.429 [-1.106,1.964] | 1.562^*^ [0.215,2.909] | 0.011^**^ [0.003,0.020] | 0.164 [-0.522,0.851] | 0.227^*^ [0.031,0.423] | 0.867 [-0.291,2.025] | -0.026^*^ [-0.049,-0.002] |
| **Marital status (ref=married)** |  |  |  |  |  |  |  |  |  |  |  |
| Single | 0.989 [0.974,1.005] | -0.277^***^ [-0.398,-0.157] | 0.052^*^ [0.005,0.099] | 1.146 [-0.237,2.529] | -3.083^***^ [-4.237,-1.930] | 0.169 [-0.960,1.297] | -0.011^**^ [-0.018,-0.004] | -1.006^***^ [-1.545,-0.468] | -0.105 [-0.262,0.053] | -1.358^**^ [-2.265,-0.451] | -0.044^***^ [-0.063,-0.026] |
| Divorced | 1.012 [0.992,1.032] | 0.138^*^ [0.005,0.270] | 0.045 [-0.007,0.097] | 2.214^*^ [0.383,4.045] | 1.992^**^ [0.677,3.306] | 2.005^***^ [0.882,3.127] | 0.008^*^ [0.000,0.016] | 0.657^*^ [0.049,1.265] | 0.148 [-0.037,0.333] | -2.359^***^ [-3.352,-1.367] | -0.071^***^ [-0.097,-0.045] |
| Widowed | 0.998 [0.971,1.025] | -0.178 [-0.367,0.011] | 0.013 [-0.056,0.082] | 3.170^*^ [0.685,5.656] | -3.242^***^ [-4.839,-1.645] | -0.726 [-2.097,0.646] | -0.013^**^ [-0.021,-0.004] | -0.852^*^ [-1.527,-0.176] | -0.161 [-0.388,0.067] | -1.537^**^ [-2.687,-0.388] | -0.053^***^ [-0.085,-0.022] |
| **Social class (ref=Managerial & professional)** |  |  |  |  |  |  |  |  |  |  |  |
| Intermediate | 1.005 [0.990,1.021] | -0.107 [-0.217,0.003] | -0.038 [-0.084,0.009] | 0.672 [-0.791,2.135] | 0.625 [-0.514,1.765] | 1.283^*^ [0.218,2.348] | 0.009^**^ [0.003,0.015] | 0.395 [-0.106,0.895] | 0.083 [-0.071,0.236] | -0.030 [-0.767,0.706] | -0.017^*^ [-0.032,-0.002] |
| Routine and manual | 1.006 [0.992,1.020] | -0.157^**^ [-0.270,-0.045] | -0.071^***^ [-0.112,-0.030] | 1.091 [-0.192,2.374] | 0.880 [-0.165,1.925] | 1.230^*^ [0.279,2.181] | 0.014^***^ [0.007,0.021] | 0.556^*^ [0.064,1.049] | 0.260^***^ [0.122,0.397] | -0.648 [-1.402,0.105] | -0.045^***^ [-0.060,-0.029] |
| **Mental disorder/stigma group (ref=No MD/less stigmatising attitudes)** |  |  |  |  |  |  |  |  |  |  |  |
| No MD/more stigmatising attitudes | 1.000 [0.986,1.014] | 0.057 [-0.050,0.163] | 0.038 [-0.003,0.080] | 0.567 [-0.730,1.863] | 0.244 [-0.756,1.244] | -0.496 [-1.445,0.452] | 0.001 [-0.005,0.006] | 0.099 [-0.327,0.525] | 0.037 [-0.102,0.176] | -1.384^***^ [-2.107,-0.661] | -0.002 [-0.016,0.013] |
| CMD/less stigmatising attitudes | 1.017^*^ [1.000,1.033] | 0.073 [-0.035,0.181] | -0.068^***^ [-0.109,-0.028] | -0.234 [-1.593,1.124] | 0.478 [-0.497,1.453] | 1.178^*^ [0.199,2.157] | 0.010^**^ [0.004,0.016] | 1.168^***^ [0.685,1.650] | 0.269^***^ [0.125,0.413] | -4.661^***^ [-5.426,-3.895] | -0.106^***^ [-0.126,-0.086] |
| CMD/more stigmatising attitudes | 1.011 [0.991,1.031] | 0.247^**^ [0.089,0.405] | -0.018 [-0.082,0.046] | -2.190^*^ [-4.286,-0.094] | 0.180 [-1.375,1.734] | 0.605 [-0.749,1.959] | 0.009 [-0.001,0.019] | 0.760 [-0.047,1.567] | 0.221 [-0.016,0.458] | -5.798^***^ [-6.988,-4.608] | -0.127^***^ [-0.161,-0.092] |
| SMI/less stigmatising attitudes | 1.023 [0.985,1.062] | 0.174 [-0.065,0.414] | -0.040 [-0.121,0.041] | -1.716 [-4.678,1.247] | 0.291 [-2.004,2.586] | 1.553 [-0.522,3.629] | 0.018^*^ [0.003,0.034] | 0.705 [-0.334,1.743] | 0.475^*^ [0.098,0.852] | -8.670^***^ [-10.478,-6.862] | -0.238^***^ [-0.294,-0.183] |
| SMI/more stigmatising attitudes | 1.042 [0.986,1.101] | -0.072 [-0.357,0.213] | 0.019 [-0.150,0.189] | -2.295 [-6.792,2.202] | 2.824 [-0.965,6.612] | 2.393 [-1.215,6.001] | 0.011 [-0.011,0.033] | 0.024 [-1.802,1.850] | 0.069 [-0.524,0.661] | -10.312^***^ [-13.554,-7.070] | -0.232^***^ [-0.325,-0.138] |
| N | 3555 | 3429 | 3559 | 4239 | 4239 | 4875 | 4762 | 4605 | 3680 | 4792 | 4871 |

^a^ Exponentiated coefficient

BMI=Body Mass Index; CI=confidence interval; CMD=common mental disorder; Coeff.=unstandardised coefficient; EQ-5D=EuroQol-5D; GCSE=General Certificate of Secondary Education; HDL=high density lipoprotein; MD=mental disorder; N=number of individuals; Ref=reference category; SMI=severe mental illness; WEMWBS=Warwick-Edinburgh Mental Well-Being Scale

^*^ *p* < 0.05, ^**^ *p* < 0.01, ^***^ *p* < 0.001

Table A.3: Results from linear regression models assessing the association between mental disorder/stigma group and health and wellbeing outcomes for the measure of prejudice and exclusion

|  | **Glycated haemoglobin**^a^ | **Total cholesterol (mmol/L)** | **HDL cholesterol (mmol/L)** | **Systolic blood pressure (mmHg)** | **Diastolic blood pressure (mmHg)** | **Resting pulse rate (bpm)** | **Waist-hip ratio** | **BMI (kg/m^2^)** | **Allostatic load score** | **WEMWBS** | **EQ-5D** |
| --- | --- | --- | --- | --- | --- | --- | --- | --- | --- | --- | --- |
|  | Coeff.  [95% CI] | Coeff.  [95% CI] | Coeff.  [95% CI] | Coeff.  [95% CI] | Coeff.  [95% CI] | Coeff.  [95% CI] | Coeff.  [95% CI] | Coeff.  [95% CI] | Coeff.  [95% CI] | Coeff.  [95% CI] | Coeff.  [95% CI] |
| **Gender (ref=male)** |  |  |  |  |  |  |  |  |  |  |  |
| Female | 0.997 [0.985,1.008] | 0.046 [-0.039,0.131] | 0.280^***^ [0.249,0.311] | -8.209^***^ [-9.229,-7.190] | -1.275^***^ [-2.023,-0.527] | 2.895^***^ [2.150,3.640] | -0.094^***^ [-0.098,-0.090] | -0.188 [-0.515,0.138] | -0.089 [-0.197,0.019] | 0.261 [-0.295,0.817] | -0.012 [-0.024,0.000] |
| **Age** | 1.004^***^ [1.003,1.004] | 0.014^***^ [0.011,0.017] | 0.003^***^ [0.001,0.004] | 0.427^***^ [0.391,0.464] | 0.102^***^ [0.075,0.130] | -0.029^*^ [-0.055,-0.003] | 0.002^***^ [0.002,0.002] | 0.055^***^ [0.042,0.068] | 0.032^***^ [0.028,0.035] | 0.022^*^ [0.001,0.043] | -0.002^***^ [-0.003,-0.002] |
| **Education level (ref=degree)** |  |  |  |  |  |  |  |  |  |  |  |
| A Level | 1.013 [0.999,1.027] | 0.006 [-0.106,0.118] | -0.083^***^ [-0.125,-0.041] | 1.677^*^ [0.274,3.081] | 0.223 [-0.862,1.307] | 0.246 [-0.795,1.286] | 0.007^*^ [0.001,0.014] | 0.323 [-0.131,0.778] | 0.231^**^ [0.093,0.370] | -0.515 [-1.267,0.237] | -0.008 [-0.023,0.006] |
| GCSE | 1.030^***^ [1.014,1.046] | 0.062 [-0.061,0.184] | -0.097^***^ [-0.145,-0.048] | 0.891 [-0.594,2.376] | -0.410 [-1.598,0.779] | 0.930 [-0.203,2.063] | 0.012^**^ [0.004,0.019] | 1.153^***^ [0.620,1.685] | 0.361^***^ [0.194,0.528] | -1.655^***^ [-2.475,-0.834] | -0.033^***^ [-0.049,-0.016] |
| None | 1.038^***^ [1.019,1.058] | -0.178^*^ [-0.325,-0.031] | -0.101^**^ [-0.162,-0.041] | 1.449 [-0.414,3.313] | -2.984^***^ [-4.355,-1.613] | 1.258 [-0.047,2.564] | 0.013^**^ [0.004,0.021] | 0.730^*^ [0.070,1.391] | 0.252^*^ [0.059,0.446] | -2.687^***^ [-3.726,-1.649] | -0.071^***^ [-0.093,-0.049] |
| **Ethnicity (ref=white)** |  |  |  |  |  |  |  |  |  |  |  |
| Non-white | 1.065^***^ [1.037,1.095] | -0.071 [-0.250,0.109] | -0.087^**^ [-0.149,-0.025] | -2.773^**^ [-4.645,-0.901] | 0.543 [-1.036,2.123] | 1.615^*^ [0.228,3.003] | 0.013^**^ [0.004,0.021] | 0.276 [-0.427,0.980] | 0.267^**^ [0.068,0.467] | 0.906 [-0.266,2.077] | -0.025^*^ [-0.048,-0.001] |
| **Marital status (ref=married)** |  |  |  |  |  |  |  |  |  |  |  |
| Single | 0.990 [0.975,1.006] | -0.284^***^ [-0.404,-0.165] | 0.054^*^ [0.006,0.101] | 1.155 [-0.224,2.533] | -3.070^***^ [-4.220,-1.921] | 0.174 [-0.957,1.304] | -0.012^**^ [-0.019,-0.005] | -1.023^***^ [-1.557,-0.488] | -0.118 [-0.275,0.039] | -1.413^**^ [-2.321,-0.505] | -0.044^***^ [-0.062,-0.026] |
| Divorced | 1.011 [0.991,1.032] | 0.144^*^ [0.010,0.278] | 0.045 [-0.008,0.097] | 2.214^*^ [0.388,4.040] | 1.999^**^ [0.690,3.307] | 2.003^***^ [0.880,3.125] | 0.008^*^ [0.000,0.016] | 0.672^*^ [0.063,1.280] | 0.154 [-0.030,0.338] | -2.350^***^ [-3.354,-1.347] | -0.071^***^ [-0.097,-0.044] |
| Widowed | 0.997 [0.971,1.025] | -0.169 [-0.358,0.020] | 0.016 [-0.053,0.085] | 3.173^*^ [0.680,5.665] | -3.172^***^ [-4.779,-1.566] | -0.717 [-2.086,0.652] | -0.012^**^ [-0.021,-0.004] | -0.815^*^ [-1.488,-0.143] | -0.144 [-0.373,0.085] | -1.524^**^ [-2.682,-0.366] | -0.053^***^ [-0.085,-0.022] |
| **Social class (ref=Managerial & professional)** |  |  |  |  |  |  |  |  |  |  |  |
| Intermediate | 1.005 [0.989,1.020] | -0.097 [-0.207,0.013] | -0.035 [-0.082,0.012] | 0.727 [-0.727,2.181] | 0.686 [-0.449,1.821] | 1.266^*^ [0.204,2.329] | 0.009^**^ [0.003,0.016] | 0.428 [-0.074,0.931] | 0.096 [-0.058,0.250] | -0.076 [-0.814,0.662] | -0.017^*^ [-0.032,-0.002] |
| Routine and manual | 1.005 [0.992,1.019] | -0.144^*^ [-0.255,-0.032] | -0.068^**^ [-0.109,-0.027] | 1.111 [-0.177,2.399] | 0.967 [-0.070,2.004] | 1.236^*^ [0.287,2.185] | 0.015^***^ [0.008,0.021] | 0.611^*^ [0.126,1.096] | 0.281^***^ [0.143,0.418] | -0.678 [-1.432,0.075] | -0.044^***^ [-0.060,-0.029] |
| **Mental disorder/stigma group (ref=No MD/less stigmatising attitudes)** |  |  |  |  |  |  |  |  |  |  |  |
| No MD/more stigmatising attitudes | 1.003 [0.986,1.020] | -0.053 [-0.173,0.066] | 0.002 [-0.043,0.046] | 0.362 [-1.110,1.833] | -0.456 [-1.536,0.623] | -0.487 [-1.501,0.526] | -0.005 [-0.011,0.001] | -0.380 [-0.819,0.060] | -0.151^*^ [-0.295,-0.006] | -0.688 [-1.558,0.183] | -0.002 [-0.017,0.014] |
| CMD/less stigmatising attitudes | 1.014 [0.999,1.029] | 0.097 [-0.004,0.199] | -0.072^***^ [-0.112,-0.032] | -0.146 [-1.447,1.155] | 0.534 [-0.399,1.467] | 1.088^*^ [0.164,2.011] | 0.008^**^ [0.002,0.015] | 1.131^***^ [0.657,1.606] | 0.239^***^ [0.101,0.378] | -4.373^***^ [-5.101,-3.645] | -0.103^***^ [-0.122,-0.084] |
| CMD/more stigmatising attitudes | 1.026^*^ [1.002,1.051] | 0.003 [-0.204,0.211] | -0.052 [-0.118,0.014] | -3.608^**^ [-5.874,-1.343] | -1.225 [-2.872,0.421] | 1.048 [-0.663,2.759] | 0.006 [-0.004,0.016] | 0.076 [-0.737,0.889] | 0.051 [-0.227,0.329] | -5.897^***^ [-7.302,-4.493] | -0.147^***^ [-0.189,-0.105] |
| SMI/less stigmatising attitudes | 1.035 [0.998,1.074] | 0.038 [-0.169,0.244] | -0.045 [-0.131,0.042] | -2.560 [-5.397,0.277] | 0.056 [-2.215,2.327] | 2.067 [-0.016,4.151] | 0.013 [-0.002,0.027] | 0.194 [-0.769,1.157] | 0.308 [-0.064,0.680] | -9.140^***^ [-10.912,-7.367] | -0.228^***^ [-0.279,-0.177] |
| SMI/more stigmatising attitudes | 1.005 [0.954,1.060] | 0.188 [-0.276,0.651] | 0.003 [-0.153,0.159] | 0.715 [-3.738,5.168] | 3.826^*^ [0.810,6.842] | 0.858 [-2.621,4.337] | 0.022 [-0.002,0.046] | 1.139 [-0.904,3.183] | 0.259 [-0.274,0.792] | -7.579^***^ [-11.262,-3.896] | -0.272^***^ [-0.389,-0.155] |
| N | 3555 | 3429 | 3559 | 4239 | 4239 | 4875 | 4762 | 4605 | 3680 | 4792 | 4871 |

^a^ Exponentiated coefficient

BMI=Body Mass Index; CI=confidence interval; CMD=common mental disorder; Coeff.=unstandardised coefficient; EQ-5D=EuroQol-5D; GCSE=General Certificate of Secondary Education; HDL=high density lipoprotein;  MD=mental disorder; N=number of individuals; Ref=reference category; SMI=severe mental illness; WEMWBS=Warwick-Edinburgh Mental Well-Being Scale

^*^ *p* < 0.05, ^**^ *p* < 0.01, ^***^ *p* < 0.001
